# Tannic Acid Induces Endoplasmic Reticulum Stress-Mediated Apoptosis in Prostate Cancer

**DOI:** 10.3390/cancers10030068

**Published:** 2018-03-07

**Authors:** Prashanth K.B. Nagesh, Elham Hatami, Pallabita Chowdhury, Vivek K. Kashyap, Sheema Khan, Bilal B. Hafeez, Subhash C. Chauhan, Meena Jaggi, Murali M. Yallapu

**Affiliations:** Department of Pharmaceutical Sciences and Center for Cancer Research, University of Tennessee Health Science Center, Memphis, TN 38163, USA; pbhusett@uthsc.edu (P.K.B.N.); ehatami@uthsc.edu (E.H.); pchowdhu@uthsc.edu (P.C.); vkashya1@uthsc.edu (V.K.K.); skhan14@uthsc.edu (S.K.); bhafeez@uthsc.edu (B.B.H.); schauha1@uthsc.edu (S.C.C.); mjaggi@uthsc.edu (M.J.)

**Keywords:** ER stress, molecularly targeted therapeutics, tannic acid, apoptosis, unfolded protein response

## Abstract

Endoplasmic reticulum (ER) stress is an intriguing target with significant clinical importance in chemotherapy. Interference with ER functions can lead to the accumulation of unfolded proteins, as detected by transmembrane sensors that instigate the unfolded protein response (UPR). Therefore, controlling induced UPR via ER stress with natural compounds could be a novel therapeutic strategy for the management of prostate cancer. Tannic acid (a naturally occurring polyphenol) was used to examine the ER stress mediated UPR pathway in prostate cancer cells. Tannic acid treatment inhibited the growth, clonogenic, invasive, and migratory potential of prostate cancer cells. Tannic acid demonstrated activation of ER stress response (Protein kinase R-like endoplasmic reticulum kinase (PERK) and inositol requiring enzyme 1 (IRE1)) and altered its regulatory proteins (ATF4, Bip, and PDI) expression. Tannic acid treatment affirmed upregulation of apoptosis-associated markers (Bak, Bim, cleaved caspase 3, and cleaved PARP), while downregulation of pro-survival proteins (Bcl-2 and Bcl-xL). Tannic acid exhibited elevated G_1_ population, due to increase in p18^INK4C^ and p21^WAF1/CIP1^ expression, while cyclin D1 expression was inhibited. Reduction of MMP2 and MMP9, and reinstated E-cadherin signifies the anti-metastatic potential of this compound. Altogether, these results demonstrate that tannic acid can promote apoptosis via the ER stress mediated UPR pathway, indicating a potential candidate for cancer treatment.

## 1. Introduction

Prostate cancer is the second leading cause of cancer related death in men in the United States [1]. According to Cancer Statistics, in 2018, it accounts for 19% of cancer incidences in men among all of the cancers in the United States [1]. It is estimated that there will be 164,690 new cases of prostate cancer diagnosed in 2018 with conservative projections of 29,430 deaths due to this disease. Current clinical treatment consists of both single and combination chemotherapy along with radiation and surgical procedures. Clinically, polychemotherapy offers a better survival advantage when compared to other therapies. However, empirically designed drugs in combination may not be effective due to distinctly different mechanisms of action, pharmacokinetics, and pharmacodynamic profiles of the individual drugs. In general, combination drug regimens have a smaller therapeutic window, making the patient’s individual response to therapy an important consideration when navigating drug therapy options [2,3]. Additionally, such drug combination treatment results in severe systemic toxicity. 

Endoplasmic reticulum (ER) is a crucial organelle that is responsible for several fundamental cellular activities, including synthesis, folding, maturation, and translocation of intracellular proteins. The ER serves as the principal site for synthesis and the folding of proteins in cells [4]. ER stress leads to accumulation of unfolded or misfolded proteins that are removed by proteolytic mechanisms. Often, these proteins follow ubiquitin/proteasome and autophagy/lysosomal mediated degradation [5]. Disturbances to ER homeostasis i.e., accumulation of unfolded/aggregated proteins promote an ER adaptation capacity, which is known as unfolded protein response (UPR). Such UPR activation has been implicated in several metastasic cancers [6]. The UPR phenomenon is mainly a pro-survival process but prolonged and severe ER stress results in significant protein accumulation and will induce a UPR that will lead to cell death. The mechanisms regulating the cell’s survival/death decision under ER stress might be crucial in order to target tumor cells and overcome tumor resistance during therapies [7]. Protein kinase R-like endoplasmic reticulum kinase (PERK), activating transcription factor 6 (ATF6), and inositol requiring enzyme 1 alpha (IRE1α) are three branches in the ER lumen that are considered to be ER stress sensor proteins [7]. Binding immunoglobulin protein (BiP) (also known as 78 kDa glucose-regulated protein, GRP-78) is a master regulator of ER function and a key molecule responsible for inducing UPR and cancer cell survival [8]. The up-regulated expression of Bip is known to cause activation of ER stress. Chronic ER stress activates UPR in the ER lumen leading to severe stress in cells which often induces apoptosis in cancer cells [7]. Thapsigargin is a potent and specific cell-permeable inhibitor of the ER Ca^2+^-ATPase [9]. Thapsigargin treatment resulted in reduced ER calcium levels that offer lowered chaperone activity. This promotes the accumulation of unfolded proteins in ER lumen. Thus, thapsigargin is considered as a positive ER stress inducer (like tunicamycin, Brefedin A, dithiothreitol, and MG132) which activates the UPR [9]. Further, prolonged or severe ER stress triggers cell death by cross-talk with the intrinsic or extrinsic apoptotic pathways [10]. If a small molecule stimulates ER stress, it may not only induce cancer cell apoptotic mechanisms but also inhibit the tumor growth, migration, and invasion. Epithelial to Mesenchymal Transition (EMT) is the primordial event in the cancer progression and metastasis; in which tumorigenic cells transform in mesenchymal phenotypes [11]. Therefore, in the therapeutic perspective, the induction of ER stress could be an attractive molecular target in cancer therapies. 

Dietary compounds, such as phenolics and flavonoids, have been validated to display anti-cancer activity and reduced the risk of prostate cancer [12,13,14]. Also, curcumin, quercetin, rutin, morin, resveratrol, gallic acid, (phenolics of natural origin), and so on were demonstrated to inhibit proliferation of prostate cancer cells [15,16]. Other phenolic and flavonoid compounds, such as ellagic acid and apigenin, have been demonstrated to inhibit and prevent cancer and its recurrence of various malignancies [17,18]. Tannic acid (TA) is a naturally occurring polyphenolic compound that is widely found in plant seeds/leaves, fruit skins, and wood bark. Studies suggest that this molecule exhibits chemopreventive, chemosensitization, and antitumor properties [19]. Tannic Acid is commonly used as a food, drink, and pharmaceutical additive. More importantly, TA is recognized as a safe compound by the US Food and Drug Administration (See 9 CFR 318.7, FDA food additive list) [20]. Thus, tannic acid is commonly used in beer clarification, soft drinks and juices, especially to enhance taste and color stabilization in wine [21]. Tannic acid is a hydrolysable phenolic molecule, containing a central glucose unit and gallic acid esterified to it. The hydroxyl and carboxyl functional groups in TA allows for binding proteins and DNA, making TA an important candidate for antimicrobial and antifungal agents [22,23]. Chemopreventive and chemosensitization activity of TA has been demonstrated against several human carcinomas such as breast, ovarian and skin cancers [24,25,26,27]. Literature suggests that TA can inhibit various oncogenic signaling cascades [28,29]. However, TA’s influence on induced ER stress mediated UPR signaling has never been investigated. Thus, in this study, we aim to elucidate these effects of TA for its ability to reduce risk in prostate cancer cells. The results of this study demonstrate that TA inhibited proliferation, invasion, and migration of human advanced prostate cancer cells via inducing ER stress modulation.

## 2. Results

### 2.1. Tannic Acid Treatment Suppresses Proliferation and Clonogelnicity of Prostate Cancer Cells

**Figure 1:**
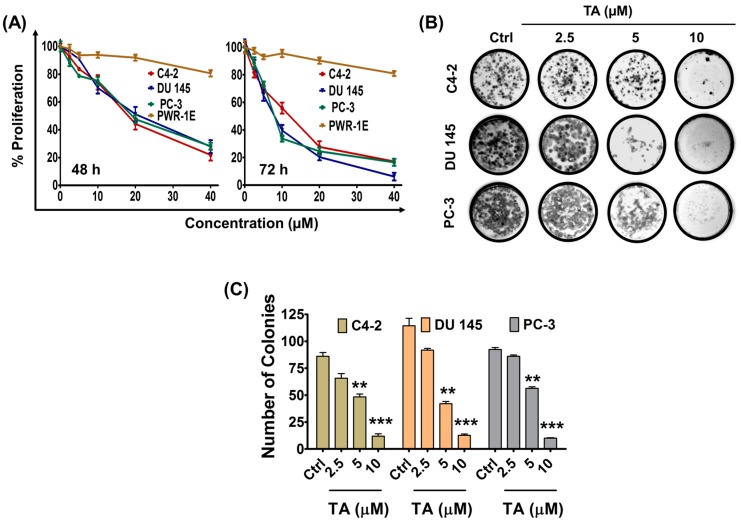
Tannic acid inhibited the growth of prostate cancer cells. (**A**) Effect of Tannic acid (TA) on cell proliferation of human prostatic epithelial cells (PWR1E) and prostate cancer (C4-2, DU 145 and PC-3) cells. Cells (5000) were seeded in each well of 96-well plate and allowed to grow overnight, the cells were then treated with the described concentrations for 48 and 72 h. The line graphs represent the percent proliferation compared with the vehicle-treated group cells. Data indicated TA is not toxic to PWR1E cells; (**B**) Effect of TA on clonogenic potential of prostate cancer cells. Representative colony images of control and TA treated C4-2, DU 145, and PC-3 cells; and, (**C**) Bar graphs indicating quantification of colony formation in C4-2, DU 145 and PC-3. Data represent the mean of triplicates ± SEM, ** *p* < 0.01, and *** *p* < 0.001.

Tannic acid acts as a chemopreventing and anti-cancer agent in various cancers. Herein, we tested its cytotoxicity against prostate cancer cells. The cytotoxic effects of serial concentrations of TA (for 48 and 72 h treatment) on prostate cancer cells were examined using the MTS assay (Figure 1A). Notably, TA treatment caused a dose dependent (1.25–40 μM) decrease in viability of C4-2, DU 145 and PC-3 cells. The IC_50_ (the concentration of TA where the cells growth is reduced by half) in all prostate cancer cells was achieved within a concentration range of 20–25 μM for 48 h and 9–13 μM for 72 h treatment. In detail, IC_50_ values were calculated as: 20.80 ± 1.50, 24.52 ± 1.95, and 23.61 ± 1.74 μM (48 h treatment), and 12.92 ± 1.18, 8.95 ± 0.80, 8.53 ± 0.80 (72 h treatment) in C4-2, DU 145, and PC-3 cells, respectively. However, we did not observe any significant effect of TA on PWR-1E normal prostate epithelial cells. Next, the effect of TA treatment was examined on colony inhibition of prostate cancer cells (Figure 1B,C). Although a colony formation assay is time-consuming, it is considered the gold standard of in vitro assays for testing the activity of compounds and to test long term growth of cell lines [30]. TA treatment inhibited formation of colonies in a (2.5–10 μM) dose-dependent manner. These results demonstrate that TA efficiently inhibited the growth and clonogenicity of prostate cancer cells. TA effects are very similar in all three prostate cancer cell lines (C4-2, DU 145, and PC-3) tested. Furthermore, PC-3 and DU 145 cell lines exhibits similar phenotypic characteristics. Therefore, we have chosen C4-2 and PC-3 cell lines for further functional studies, such as gene and protein profiling studies.

### 2.2. Tannic Acid Treatment Upregulates the Expression of ER Stress Regulatory Proteins

The ER stress pathway is considered a novel pathway of interest in regards to the development of cancer therapeutic agents [7]. Since TA inhibited proliferation of prostate cancer cells, we examined the involvement of TA in ER stress pathway in prostate cancer cells. To test the ER associated stress pathway, we examined the activation of ER stress marker sensors and their downstream signaling molecules.

**Figure 2:**
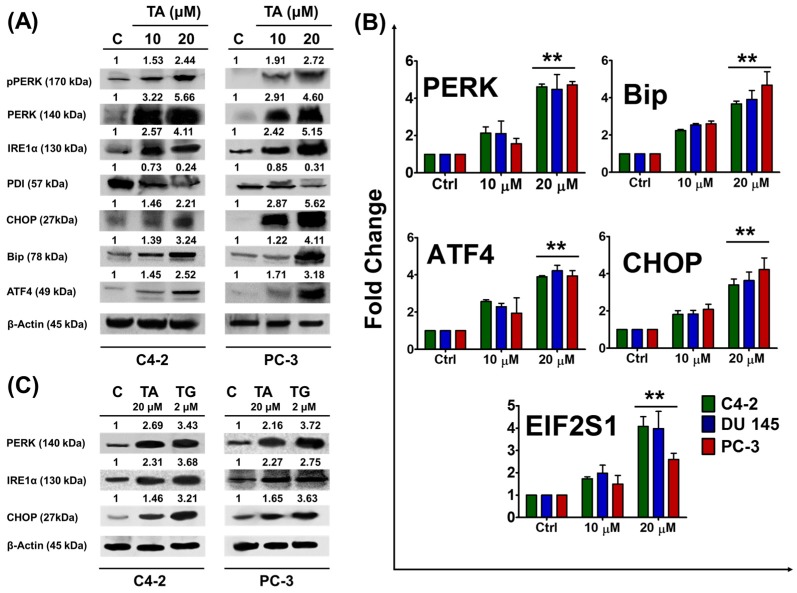
Tannic acid induced ER stress in prostate cancer cells. (**A**) Western blot analysis of Protein kinase R-like endoplasmic reticulum kinase (PERK), inositol requiring enzyme 1 alpha (IRE1α), and activating transcription factor 4 (ATF4) signaling in prostate cancer cells after dose-dependent treatment with TA. Briefly, cells were treated with indicated concentrations of TA, protein extracts were prepared and subjected for western blot analysis to detect the protein levels. β-Actin antibody served as an internal control; (**B**) Gene expression studies endoplasmic reticulum (ER) stress markers in TA treated cells for *PERK*, eukaryotic translation initiation factor 1 *(EIF2S1*), binding immunoglobulin protein (*BiP*), transcription factor C/EBP, homologous protein *(CHOP*), and *ATF4* determined by qRT-PCR analysis. GAPDH was used as an internal control. Data represent the mean of triplicates ± SEM, ** *p* < 0.01; (**C**) Cells were grown and exposed to TA and Thapsigargin. Western blot analysis of regulatory protein expression of ER stress in TA and thapsigargin (TG) treated C4-2 and PC-3 cells.

Tannic acid treatment (24 h) resulted in the dose dependent expression of Bip protein in prostate cancer cells as compared to the control, as determined by Western blot analysis (Figure 2A). Tannic acid treatment efficiently induced the expression of these sensor proteins (PERK and IRE1α) as compared to the control group (Figure 2A). The activation of the PERK protein by TA treatment prompted us to examine the downstream UPR target proteins, activating transcription factor 4 (ATF4) and transcription factor C/EBP, homologous protein (CHOP). Interestingly, both ATF4 and CHOP proteins were significantly elevated with TA treatment. In addition, TA is very effective in inducing these protein expressions at the mRNA levels (Figure 2B) (primers used for this experiment was shown in Table 1). These results found elevated the expressions of PERK (2.13 ± 0.33, 2.11 ± 0.67, 1.56 ± 0.28 and 4.62 ± 0.14, 4.49 ± 0.80, 4.72 ± 0.18 fold), Bip (2.25 ± 0.05, 2.54 ± 0.07, 2.61 ± 0.14 and 3.68 ± 0.14, 3.91 ± 0.48, 4.67 ± 0.73 fold), ATF4 (2.56 ± 0.10, 2.29 ± 0.17, 1.94 ± 0.82 and 3.89 ± 0.06, 4.22 ± 0.29, 3.94 ± 0.28 fold), CHOP (1.81 ± 0.20, 1.83 ± 0.20, 2.08 ± 0.27 and 3.40 ± 0.31, 3.63 ± 0.46, and 4.22 ± 0.62 fold), and eukaryotic translation initiation factor 1 (EIF2S1) (1.73 ± 0.08, 1.98 ± 0.35, 1.49 ± 0.38, and 4.07 ± 0.43, 3.97 ± 0.77, and 2.59 ± 0.27 fold), during TA 10 and 20 µM treatments with respect to control in C4-2, DU 145, and PC-3, respectively. 

To confirm the effectiveness and validate the central role of TA (20 µM) as an ER stress inducer, we further quantified the expression of PERK, IRE1α, and CHOP at the protein through Western blot and compared that data with thapsigargin (2 µM, a positive reference control) (Figure 2C). Tannic acid is very effective in inducing these proteins indicating their roles in inducing ER stress. Additionally, these results were further evaluated at mRNA level through q-PCR (Figure S1). 

### 2.3. TA Treatment Effectively Arrests the Cell Cycle and Modulates Cell Cycle Regulatory Proteins 

**Figure 3:**
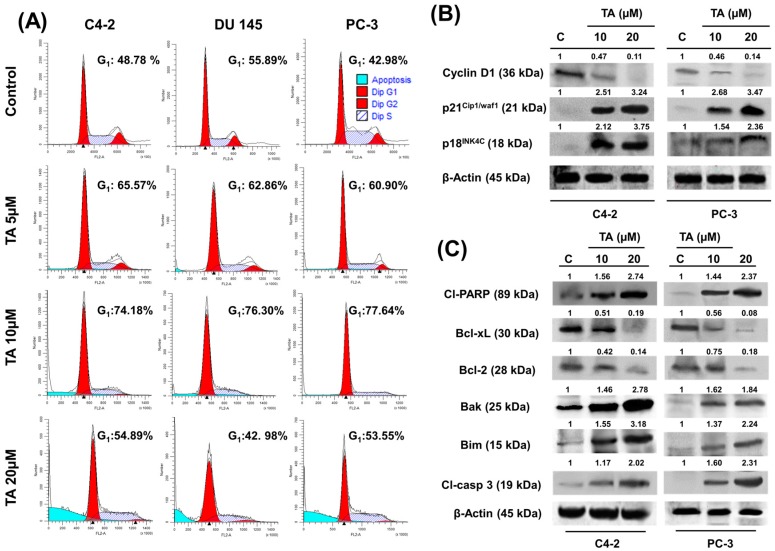
Tannic acid induces G_1_ phase arrest and apoptosis in prostate cancer cells. Prostate cancer cells were treated with TA for 24 h and cell cycle analysis was performed by flow cytometer. (**A**) Histogram plot of C4-2, DU 145 and PC-3 cells after treatment of 5, 10, and 20 µM TA. Untreated cells were used as control; (**B**) Directive role of TA on cell cycle regulatory proteins and its effect on G_1_ phase arrest. Western blot analysis of G_1_ phase cell cycle regulatory proteins in prostate cancer cells treated with TA. Tannic acid treated cell lysates were prepared and subjected for Western blot analysis. β-actin was probed for equal protein loading in each lane; and, (**C**) Cells treated with TA 10 and 20 µM for 24 h, cell lysates were collected and immunoblotted for apoptotic protein expressions.

Previous studies have demonstrated that ER stress triggers G_1_-phase cell cycle arrest in various cancer cells [31]. Various polyphenols showed cytostatic effects on prostate cancer cells [32]. Therefore, we investigated the cell cycle analysis using PI/RNase staining solution, employing flow cytometry. Tannic acid treatment (5 and 10 μM) after 24 h demonstrated an increase in the G_1_ population of prostate cancer cells (Figure 3A) as compared to the untreated control cells. The percentage of G_1_ phase increased: from 48.78% (C4-2 control cells) to 65.57% and 74.18%; 55.89% (DU 145 control cells) to 62.86% and 76.30%; 42.98% (PC-3 control cells) to 60.90% and 77.64%; after treatment with 5 and 10 µM TA, respectively. In addition, 20 μM TA treatment induced a prominent rise in apoptotic populations, as observed (Figure 3A). To further investigate the molecular mechanism underlying cell cycle arrest, we examined the effect of TA treatment on cell cycle regulatory proteins (cell cycle inhibitory proteins (p21^INK4C^ and p18^INK4C^) and cyclin D1) in prostate cancer cells. Western blot analysis demonstrated that TA treatment significantly increased the protein levels of p21^WAF1/CIP1^ and p18^INK4C^, while it decreased the protein levels of cyclin D1 (G_1_ phase positive regulator) in prostate cancer cells when compared to control cells (Figure 3B). 

### 2.4. Tannic Acid Treatment Induced Apoptosis via ER Stress Mediated Signaling

To investigate the mechanistic role of TA in apoptosis, we sought to examine the expression of key proteins that are associated in cell proliferation and the survival process. This data suggests a dose-dependent decrease in the expression of anti-apoptotic proteins (Bcl-xL and Bcl-2), while a simultaneous increase in the expression of pro-apoptotic proteins (Bim and Bak) with 24 h TA treatment. It is known that the activation of PERK and IRE1α correlates with those of decreased expression of Bcl-2 and Bcl-xL. Cleaved caspase-3 and cleaved PARP appearance further supports the apoptosis role of TA (Figure 3C).

### 2.5. Tannic Acid Treatment Inhibited Migratory and Invasive Potentials of Prostate Cancer Cells 

**Figure 4:**
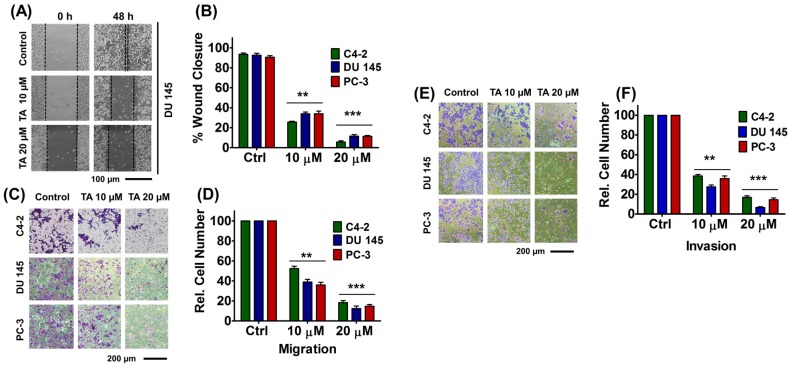
Tannic acid inhibited migratory and invasive attributes of prostate cancer cells. (**A**) Wound healing assay. In cell migration assay, a uniform scratch was made in 80% confluent monolayer cultures of prostate cancer cells and the extent of closure was monitored in presence of TA (10 and 20 µM) under phase-contrast microscopy and imaged at 0 and 48 h at 20× magnification; (**B**) Percent wound closure with TA treatment in C4-2, DU 145 and PC-3 cells; (**C**) Boyden chamber assays of TA treated prostate cancer cells. Cells were imaged under phase-contrast microscopy at 20× magnification; (**D**) Bar graph displaying relative cell number of migrated cells (per unit area) in TA-treated groups; (**E**) Matrigel Invasion assays of TA treated prostate cancer cells; and, (**F**) Bar graph displaying relative cell number of cells invaded (per unit area) in TA-treated groups. Data represent the mean of triplicates ± SEM, ** *p* < 0.01 and *** *p* < 0.001.

We determined the effects of TA on migration (wound healing i.e., scratch assay and Boyden chamber assay) and invasion (matrigel invasion) assays in prostate cancer cells. Upon treatment, the wound closure of the scratch was measured as reported previously [33]. In this assay, initial size of wound values were normalized to 100% in all C4-2, DU 145, and PC-3 cells. After 48 h, the wound size closure in control cells were found to be 93 ± 1.2%, 92 ± 1.85%, and 90 ± 1.45% (C4-2, DU 145, and PC-3) while 25 ± 0.88%, 34 ± 1.73%, and 34 ± 2.64% (10 µM of TA) and 5 ± 1.21%, 11 ± 1.45%, and 11 ± 0.89% (20 µM of TA) in C4-2, DU 145, and PC-3 cells, respectively (Figure 4A,B). 

Similarly, the anti-migratory potential of TA against C4-2, DU 145, and PC-3 cells was ratified through Boyden chamber studies. The graphical representation of base cell numbers of C4-2, DU 145, and PC-3 cells after TA treatment during migration and invasion studies were shown in Figure S2. This assay demonstrated a significant decreased 52.33 ± 2.33%, 39 ± 2.64%, and 36 ± 2.24% (C4-2, DU 145, and PC-3) migration of cells with 10 µM TA treatment while 20 µM TA treatment resulted in almost negligible migration, 18.33 ± 2.02%, 12.33 ± 2.6%, and 14.66 ± 1.76% (C4-2, DU 145, and PC-3) (Figure 4C,D). Overall, these results confirm that TA has effective anti-migratory ability. 

To examine the effect of TA on the invasive capability of prostate cancer cells, a matrigel invasion assay was employed. It was clear that after the cells were treated with TA, the invasive potential of the cells drastically diminished in comparison to the untreated cells (Figure 4E). The relative number of invaded cells was measured and graphically represented (Figure 4F). These data indicate that the relative number of invaded cells was reduced to less than half of its number, 38.66 ± 1.45%, 27.67 ± 1.76%, and 36 ± 2.64% even with 10 μM TA treatment in C4-2, DU 145, and PC-3 cells when compared with non-treated controls. Furthermore, this effect is much more significant with 20 µM treatment at 24 h (17 ± 1.52%, 6.66 ± 0.88%, and 14.66 ± 1.76%). Altogether, the TA annihilated the invasive attributes of prostate cancer cells. 

**Figure 5:**
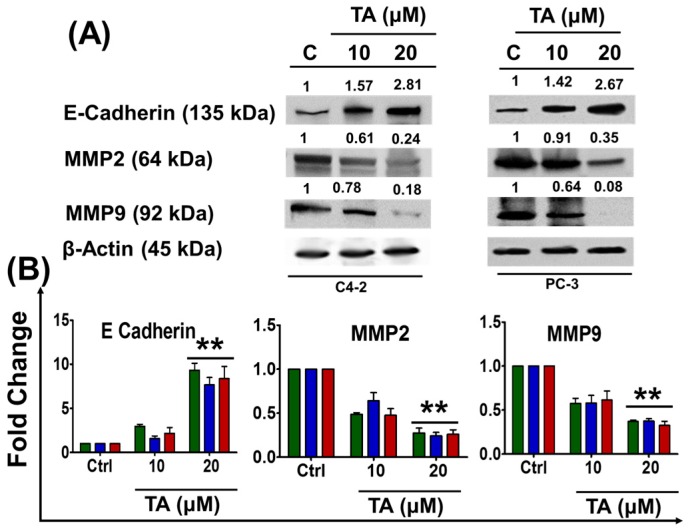
Tannic acid altered the EMT regulatory protein markers in prostate cancer cells. (**A**) Western analysis of Epithelial to Mesenchymal Transition (EMT) markers in TA treated cells. Values shown above the blots are the densitometry analysis of each protein band normalized with respective β-actin value; (**B**) Gene expression studies of EMT markers, such as E-Cadherin, MMP2 and MMP9 in TA-treated cells. Data represent the mean of triplicates ± SEM, ** *p* < 0.01.

To further validate TA’s role in inhibiting migration and invasion, we assessed the expression profiles of the epithelial marker (E-cadherin) and mesenchymal markers (MMP2 and MMP9) of EMT [34] through immuno-blot studies. Tannic acid increased the expression of E-cadherin (cell-cell adherent membrane junction protein) in both C4-2 and PC-3 cells, whereas MMP2 and MMP9 proteins were inhibited in a dose-dependent manner at 24 h (Figure 5A) as compared to control cells. These results were further confirmed by qPCR analysis (Figure 5B). Tannic acid treatment (20 µM) effectively re-expressed the E-cadherin expression 9.32 ± 0.79, 7.65 ± 0.84 and 8.37 ± 1.38 fold in C4-2, DU 145, and PC-3 cells, respectively (Figure 5B). In contrast, TA treatment of 20 µM inhibited the expression of MMP2 and MMP9, the key downstream proteins that drive the EMT in prostate cancer [35]. The fold change of MMP2 was found to be 0.27 ± 0.05, 0.24 ± 04, and 0.47 ± 0. 05, while the MMP9 fold change was found to be 0.37 ± 0. 01, 0.64 ± 0. 02, and 0.32 ± 0. 04 in C4-2, DU 145, and PC-3 cells, respectively. These data indicate an apparent influence in the mRNA levels of main effectors of the EMT signaling pathway in response to TA.

**Figure 6:**
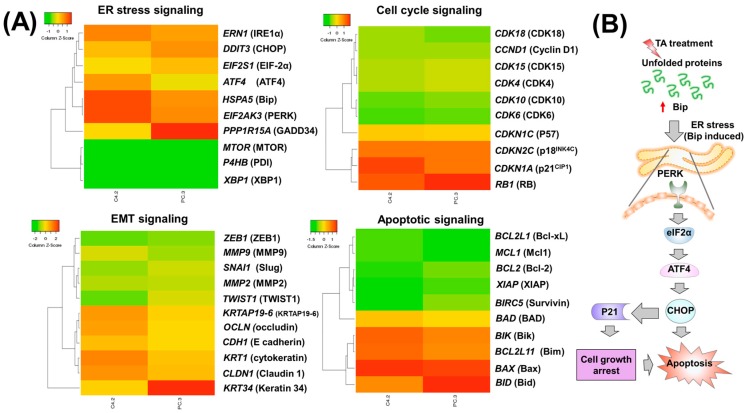
Molecular mechanism of TA in promoting unfolded protein response mediated ER stress and induced apoptosis in prostate cancer cells. (**A**) Heat map of differentially regulated genes in the ER Stress/EMT/Cell cycle regulatory/apoptosis signaling proteins in C4-2 and PC-3 prostate cancer cells; (**B**) Schematic illustration of TA-induced ER stress mediated apoptosis. .

In addition, we validated the above results of expression profiles of proteins through microarray analyses (Figure 6, Table S1) which is consistent with real time and western blot studies (Figure 2, Figure 3, Figure 4 and Figure 5). The heat maps were generated using heatmapper software (University of Alberta, Edmonton, AB, Canada) [36]. These results reflected that activation of the ER stress (PERK and IRE1α) signaling pathway could directly or indirectly contribute to TA-induced cell apoptosis (Figure 6B).

## 3. Discussion

Prostate cancer is a slow-progressing disease that requires long term care. Additionally, it is highly challenging to manage and treat with conventional regimens due to systemic toxicity, distant metastasis, and drug resistance. The outcomes and patient compliance have not improved significantly over the last few decades [37]. The quest to develop a highly specific, efficient, and safe molecule for treatment of cancers is highly desirable. Like many polyphenols (nutraceuticals), TA is commonly consumed in the form of food additives or as an excipient in many commercial drinks [38]. In addition, the FDA identified TA as a safe molecule. Tannic acid exhibited chemopreventive and anti-proliferative characteristics in a broad range of cancers by targeting multiple oncogenic signaling pathways to suppress their proliferation activity [28,29]. Also, gallic acid, a metabolite of tannic acid, was found to be ineffective towards normal epithelial cells of prostate and was consistent with our current findings [39]. However, the mechanism underlying ER stress mediated apoptotic death actions was not reported. For the first time, the present study revealed that tannic acid is proficient in inhibiting the growth of human prostate cancer cells due to its cytotoxic, cytostatic, and ER stress induction (Figure 1, Figure 2 and Figure 3). In addition, this study demonstrated the apoptotic role of TA, as well as the inhibitory effect it has on both migration and invasion of prostate cancer cells (Figure 4 and Figure 5). 

The endoplasmic reticulum (ER) is an intricate organelle that is vital for cellular function and survival. When ER activity is hindered, the accumulation of unfolded proteins stimulates the transmembrane sensors to initiate the UPR ultimately restoring ER homeostasis [40]. When the UPR fails to restore ER homeostasis and attenuates ER stress, the UPR activation induces apoptosis. Thus, measuring ER stress-mediated apoptosis will aid us [41] in developing new therapeutic options for cancer treatment. Plant based polyphenols have been reported to induce apoptosis and cell cycle arrest via induction of the ER stress mediated UPR in cancer cells. PERK and IRE1α are transmembrane proteins that are located in the ER lumen and are proximal transducers of the mammalian UPR pathway [42,43]. Initiation of ER stress leads to promoter activation of the ER stress response element found in the promoters of various UPR targets including the transcription factor CHOP, and ER chaperones like Bip [44,45]. Comparably, the induction of Bip and consistent rise in CHOP expression was observed in all prostate cancer cells during dose-dependent treatments of TA, which was further affirmed from potential studies at both gene and protein levels (Figure 2A,B). Also, activated PERK phosphorylates eIF-2α, leading to an immediate, as well as transient, protein synthesis inhibition [46]. The gene expression levels of eIF-2α (*EIF2S1*) was decreased during drug treatments, thus strengthening the rationale of TA-mediated ER stress by activation of PERK. These results manifest that TA induces ER stress-mediated UPR in prostate cancer cells.

Earlier studies suggest that during ER stress ATF4, a transcriptional regulator, translocates to the nucleus thereby inducing the expression of ER chaperones, such as protein disulfide isomerase (PDI) and various genes that are involving both antioxidant response genes and genes responsible for cellular functions like autophagy [47]. Also, PDI is an ER chaperone that is recruited during ER stress and is responsible for the formation of disulfide bonds in proteins [48]. Paradoxically, protein synthesis inhibition will induce ATF4 at the translational level, leading to activation of its downstream targets [49,50]. Comparatively, we demonstrated that the expression of PDI and ATF4 was decreased and elevated, respectively, during drug and TG treatments. We also affirmed the rise of CHOP expression, and the downstream of ATF4 during TA and TG (positive reference for ER stress) treatments at both the gene and protein levels through potential (Western) and qPCR studies (Figure 2C and Figure S1). Overexpression of BiP diminishes the CHOP induction in ER stress and reduces ER stress-induced apoptosis [51]. Research insights revealed that CHOP is implicated in programmed cell death in response to impaired ER function [52]. Therefore, the rise of CHOP during TA treatments mediates ER stress-induced apoptosis.

Mechanistic studies have shown that the overexpression/microinjection of CHOP protein into cells resulted in G_1_/S cycle arrest and/or apoptosis [53,54]. Earlier reports suggest that natural compounds of phenolic origin arrest the G_1_ phase through the ER stress pathway in lung cancer [31,55]. The decrease of eIF-2α gene expression levels causes the cessation of cyclin D1 protein synthesis, which explains the basis of G_1_ phase arrest seen in prostate cancer cells during TA treatments. Interestingly, the present study also shows that TA treatment arrests cell cycle phase distribution in association with the decreased expression of cyclin D1 and increased expression of p18^INK4C^ and p21^WAF1/CIP1^ proteins (Figure 3B). This indicates that the TA induced G_1_ arrest might be mediated through the up-regulation of p21^WAF1/CIP1^ protein, which enhances the formation of heterotrimeric complexes with G_1_-S Cdks and cyclins, thereby inhibiting its activity [56]. Our data also revealed that TA treatment reduced the protein level of Bcl-2, which might be associated with cell cycle arrest and apoptosis in prostate cancer cells, which is consistent with previous reports [57]. Gallic acid, which is a structurally similar phenolic acid, induced G_1_ phase arrest in human leukemia HL-60 cells through inhibiting cyclin D1 pathway [58], which fortifies our results. Furthermore, these findings are in accordance with earlier reports on the effect of tannic acid on breast cancer cells. Altogether, these results confirmed that TA treatment not only induced cytotoxic effects in prostate cancer cells, but also promotes the G_1_ cell cycle arrest via modulating key cell cycle regulatory proteins in prostate cancer cells.

Apoptosis is considered to be the main protective mechanism against cancer initiation and progression, otherwise cancer cells develop acquired resistance. On the other hand, the apoptotic process is controlled by both intrinsic and extrinsic pathways. An intrinsic apoptotic pathway (imbalance of pro-apoptotic and anti-apoptotic proteins) involves a caspase mediated cell death through transforming pro-caspases into active caspases. ER stress induced apoptosis utilizes this intrinsic pathway mechanism. The results indicate that TA potentiates such apoptosis machinery by inducing pro-apoptotic proteins (e.g., Bak and Bim) as well as decreased expression of anti-apoptotic proteins (Bcl-2 and Bcl-xL) (Figure 3C). Caspase-3 is known as executioner caspase, and after being activated by the initiator caspases (caspase-8), it induces apoptosis and releases both caspase 3 and PARP (cleaved caspase 3 and cleaved PARP) [59]. It is known that disruption of ER homeostasis instigates the CHOP expression along with PERK activation leading to apoptosis. CHOP is the potent regulator of apoptosis and decreases Bcl-2 protein expression [60]. We observed that TA induces the expression of CHOP which in turn inhibited anti-apoptotic proteins of the Bcl-2 family (Bcl-2 and Bcl-xL) and triggers the pro-apoptotic protein expression (Bim and Bak). Such events changes cell status from survival to apoptosis. More importantly, an induced expression of classical apoptotic protein markers (cleaved caspase 3 and cleaved PARP) suggests that TA triggers cell death in prostate cancer cells. CHOP is a transcriptional factor and is highly associated with growth arrest and apoptosis under ER stress and following DNA damage [61]. These events suggest that TA-induced apoptosis is mediated through an intrinsic mode of apoptotic signaling activation.

Tannic acid exhibits the polytrophic nature, therefore, it is possible to attenuate other cancer related pathways. Our analyses have demonstrated that TA not only regulated cell survival pathways, but is also involved in the cell cycle, cell invasion, and metastasis. Interestingly, our study has shown that TA induced apoptotic cell death and decreased cell migration and invasion (Figure 4). In another subset of experiments, we found that TA also inhibited EMT by inhibiting its key players in C4-2, DU 145, and PC-3 metastatic prostate cancer cells. We found the expression of some of the key players in EMT, such as epithelial marker (E-cadherin), was restored while mesenchymal markers (MMP2 and MMP9) were deregulated during dose-dependent TA treatments (Figure 5). Further, scratch, Boyden, and Matrigel invasion results supported and reaffirmed the anti-metastatic activity of TA and its inhibition attributes towards EMT (Figure 4). Thus, these results altogether illustrate the molecular activity of TA and its holistic mechanisms in inducing apoptosis in prostate cancer cells (Figure 6B).

Overall, this study suggests that TA is strongly correlated with ER stress and intrinsic apoptosis, which were confirmed by the expression levels of proteins. Additionally, Western blot analysis, qRT-PCR data confirm that TA regulates protein and mRNA expressions of PERK, CHOP, EIF2S1, Bip, and ATF4, proteins. These results demonstrate that TA treatment may be involved in cell growth inhibition via ER stress signaling Tannic acid might be a good candidate for combinational therapy and a highly important molecule for reducing the occurrence of prostate cancer [62]. It is well understood that ER stress can play a key role in autophagy regulation and oxidative stress. This aspect of ER stress is not evaluated in this work. However, we would consider delineating such role and validation of TA in our future studies.

## 4. Materials and Methods 

### 4.1. Materials and Reagents

All reagents, solvents, chemicals, and cell culture plastics were purchased from Sigma–Aldrich Co. (St. Louis, MO, USA) and Fisher Scientific (Pittsburgh, PA, USA), unless otherwise noted. All of the chemicals and reagents were used as received without further purification. Prostate cancer cell lines, C4-2, DU 145, and PC-3 were purchased from American Type Culture Collection (ATCC, Manassas, VA, USA) and were cultured in Roswell Park Memorial Institute medium (RPMI-1640) containing 10% (*v*/*v*) fetal bovine serum (FBS), and 1% (*w*/*v*) penicillin–streptomycin (Gibco, Thermo Fisher Scientific, Grand Island, NY, USA) and incubated at 37 °C in a humidified 5% CO_2_—95% air atmosphere (Thermo Fisher Scientific, Waltham, MA, USA). For most of the in vitro cell culture experiments, cells were trypsinized when the confluence level was about 80% and above, seeded in either 6 or 96-well plates, and were allowed to attach overnight to the plate before starting any treatments.

### 4.2. Cell Proliferation Assay 

Cell proliferation was determined using CellTiter 96^®^ AQueous One Solution Cell Proliferation Kit (MTS reagent, Promega Corporation, Madison, WI, USA) [63,64,65,66]. Briefly, 5 × 10^3^ cells in 100 μL culture medium were seeded into each well of a 96-well plate and allowed to attach overnight. Then, cells were treated with 1.25–40 µM of TA for 48 and 72 h, respectively. After completion of the treatment period, 20 μL MTS reagent was added to existing medium in the wells for 2–3 h. The intensity of the absorbed color of intracellular formazan was measured at 490 nm using a microplate reader (Cytation™ 5, BioTek Instruments, Winooski, VT, USA). The percentage of cell growth was calculated as the percentage of absorption of color intensity of the treated cells to the absorption of color intensity of untreated cells.

### 4.3. Colony Formation Assay

The effects of TA on colony inhibition of prostate cancer cells were tested by colony formation assay [65,67,68]. Briefly, cells were seeded at the density of 500 cells/well in 12-well plate and grown for two days, at 37 °C in presence of 5% CO_2_. Then, cells were treated with 2.5, 5, and 10 µM TA for seven days. On the 8th day, the medium was aspirated and replaced with fresh TA free medium containing 10% FBS. Subsequently, cells were allowed to grow till 14 days. On day 15, the cells were washed with 1X PBS and fixed using cold methanol for 1 h. After incubation, cells were washed under slow running tap water for 1 min. Then, cells were stained using 1 mL of hemotoxylin (Thermo Fisher Scientific, Waltham, MA, USA) in each well for 2 h, and then cells were further washed using tap water. The plates were dried and imaged under UVP imager BioSpectrum^®^ 500 Imaging System (UVP, Upland, CA, USA) for visualization and documentation.

### 4.4. Cell Cycle Analysis 

To study the effect of TA on the cell cycle, C4-2, DU 145, and PC-3 cells were treated with TA for 24 h after seeding in 6-well plates. After treatment, cells were trypsinized, washed with 1X PBS, and incubated in 70% ethanol; cells were kept at −20 °C overnight for fixation. The next day, cells were centrifuged, washed, and incubated with Propidium iodide (PI) solution, FxCycle™ PI/RNase Staining Solution (Thermo Fisher Scientific, Waltham, MA, USA) at 37 °C for 1 h [64,69,70]. The distribution of cells in the different cell-cycle phases was analyzed from the DNA content histogram using Accuri C6 flow cytometer (Accuri Cytometers, Inc., Ann Arbor, MI, USA) in FL-2 channel.

### 4.5. Wound Healing Assay

Effect of TA on prostate cancer cell mobility was assessed using a scratch wound assay [71,72]. For this assay, prostate cancer cells (1 × 10^6^) were cultured in a 6-well plate and after 80% confluency, the cell layer was carefully wounded using a 200 μL sterile micropipette tip. Cancer cells were treated with 10 and 20 µM TA, and the extent of drug effect was evaluated till 48 h, the residual gap length was calculated from photomicrographs.

### 4.6. Cell Migration Assay

Effect of TA on prostate cancer cell migration was evaluated through a Boyden’s chamber assay using Boyden chambers (8-μm; Corning, NY, USA) containing polycarbonate membrane [73]. Briefly, upper chamber was added with 100 μL of 5 × 10^5^ cells in serum-free medium and lower chamber was added with 250 μL of 10% FBS medium. Cells were treated with TA (10 and 20 μM) for 24 h and migrated cells into lower chamber were fixed and stained with crystal violet for 30 min at 25 °C. The migrated cells were imaged under microscope (Eclipse Ti, Nikon, Melville, NY, USA) at five random regions and the number of cells was counted to calculate the average number of migrated cells per region (ImageJ program, NIH, National Institutes of Health: Bethesda, MD, USA) (https://imagej.nih.gov/ij/) [74].

### 4.7. Cell Invasion Assay

The effects of TA on prostate cancer cell invasion were evaluated using BioCoat Matrigel Invasion Chambers (BD Biosciences, Bedford, MA, USA) that were coated with basement membrane matrix. Prostate cancer cells (3.5 × 10^4^) were seeded in the upper chamber of the trans-well containing serum free medium and cells treated with TA (10 and 20 μM) for 24 h. The tumor cells in the upper chamber were allowed to migrate into the lower chamber containing 20% FBS medium (750 μL). Cells in the top well of the upper chamber were removed by wiping them off the top membrane with cotton swabs [75]. The membranes were then fixed with 100% methanol and hematoxylin for 30 min for visualization of cells. The percentage of invasion was calculated as mentioned in migration assay.

### 4.8. cDNA Synthesis and Quantitative mRNA Expression by Real-Time PCR

Total RNA isolation of untreated and treated prostate cancer cells was performed using an RNA Isolation Kit (Qiagen Inc., Hilden, Germany). Quantity and quality of RNA were measured using NanoDrop™ 2000 (Thermo Fisher Scientific, Waltham, MA, USA). Reverse transcription of RNA to cDNA was done using cDNA Synthesis Kit (Invitrogen, Carlsbad, CA, USA). Then, cDNA was stored at −20 °C for further use [71,72]. The effect of TA on mRNA expression in prostate cancer cell lines was studied by quantitative Real Time PCR (qRT-PCR) using Roche Lightcycler 480 (Roche, Basel, Switzerland), as described previously [75]. The primer sequences (forward and reverse primers) of the genes are presented below (Table 1). Specific annealing temperatures were verified using a gradient thermal cycler. 

### 4.9. mRNA Purification, Ethanol Precipitation and Micro Array Studies

The mRNA was extracted from the TA treated C4-2 and PC-3 cells using RNA Isolation Kit (Qiagen Inc., Hilden, Germany). The extracted RNA was further purified with slight modifications [76]. To the RNA, 3 times the volume of absolute ethanol, 10% of 3M sodium acetate and 2 µL of linear acrylamide were added, mixed, vortexed, and left for incubation in −20 °C overnight. After incubation the samples were centrifuged at 130,000× *g* for 10–15 min at 4 °C. The supernatant was discarded without disturbing the pellet. 1 mL of 75–80% ethanol was added to the pellet and repeated this step. The sample was subjected to quick spin, and the ethanol was carefully removed without disturbing the pellet and it was allowed to dry, until the pellet turns opaque (avoid over drying). The pellet was suspended in 40 µL of water and vortexed down. The resultant samples were subjected to gene microarray studies using GeneChip^®^ Human gene 2.0 ST array and SensationPlusTM FFPE Amplification and WT Labeling Kit (Part# 902746) (Affymterix, Santa Clara, CA, USA).

### 4.10. Western Blot Analysis

The effects of TA on protein expression in prostate cancer cells were determined by Western blot analyses. The process of extraction, quantification of proteins, and the Western blotting method were followed, as previously described [67,77,78,79]. SDS-PAGE was performed on 4–20% gel and done using Mini-PROTEAN Tetra Cell (Bio-Rad, Richmond, CA, USA) as described earlier [67]. Proteins were transferred from gel to a nitrocellulose membrane using the trans-blot electrophoretic transfer cell containing Tris-glycine buffer, pH 8.3, and methanol. The transfer was carried out at 0–4 °C for 150 min at 55 V (400 mA) and blocked using 3% BSA for 1 h at Room temperature. Primary antibodies in 2% BSA in PBST (Bip #3177, ATF4 #111815, IRE1α #3294, PDI #3501, CHOP #2895, PERK #5683, phospho-PERK (Thr980) (16F8) #3179, Cyclin D1 #2978, p21^Cip1/waf1^ #2947, p18^INK4C^ #2896, Bcl-xL #2764, Bcl-2 #2872, Bak #3814, Bim #2819, Cleaved PARP #5625, Cleaved Caspase 3 #9664, E-cadherin #3195, MMP2 #4022, MMP9 #3852, and β-Actin #4970) (Cell Signaling Technologies, Danvers, MA, USA) were probed on a nitrocellulose membrane and were incubated at 4 °C overnight on rocker. After incubation, the blots were washed thrice with mean interval of 5 min; the membrane was further incubated with HRP conjugated secondary antibodies for 45 min. Finally, the blots were washed with PBST and bound to immunoreactive proteins on nitrocellulose membrane using Bio-Rad ECL Western Blotting Substrate Solution (Bio-Rad Laboratories, Hercules, CA, USA). Gels were imaged using a Bio-Rad computer-based gel imaging instrument and analyzed using ImageLab^TM^ software (Bio-Rad Laboratories, Hercules, CA, USA).

### 4.11. Statistical Analysis

Calculations and statistical analyses were carried out using GraphPad Prism (GraphPad Software, Inc., La Jolla, CA, USA). Mean ± SE of at least three sets of independent experiments were calculated and analyzed via two-way repeated measure analysis of variance (ANOVA) and two-tailed student’s *t*-test analysis. The level of significance was set to ** *p* < 0.01, *** *p* < 0.001.

## 5. Conclusions

Our data demonstrate the ability of TA to inhibit the growth of prostate cancer cells by arresting cell cycle in G_1_ phase. Tannic acid induced ER stress-mediated UPR was examined, and the expression of key indicators; Bip, IRE1α, and PDI were noted. The apoptotic potential of TA was determined. The downregulation of pro-survival proteins (Bcl-2 and Bcl-xL), and upregulation of pro-apoptotic markers (Bak, Bim, cleaved caspases 3) and cleaved poly (ADP-ribose) polymerase were revealed. Additionally, our results deciphered the anti-metastic activity of TA and its intriguing role in inhibiting EMT progression in prostate cancer cells. Overall, this study provides strong evidence that TA induces ER stress mediated apoptosis and cell cycle arrest. Therefore, we believe that TA may offer a novel natural monotherapy or a combination agent for prostate cancer.

## Figures and Tables

**Table 1:** Forward and reverse primer sequence of genes chosen in this study.

Genes	Forward Primers	Reverse Primers
BiP	5′-CATGGTTCTCACTAAAATGAAAGG-3′	5′-GCTGGTACAGTAACAACTG-3′
CHOP/GADD153	5′-GCCTTTCTCCTTTGGGACACTGTCCAGC-3′	5′-CTCGGCGAGTCGCCTCTACTTCCC-3′
ATF4	5′-GCATGCTCTGTTTCGAATGGA-3′	5′-CCAACGTGGTCAAGAGCTCAT-3′
PERK	5′-TCTTGGTTGGGTCTGATGAAT-3′	5′-GATGTTCTTGCTGTAGTGGGGG-3′
EIF2S1	5′-ACGACAACCCTGGAGAGAAC-3′	5′-TATGACATTCCAAGGCCGACA-3′
E-Cadherin	5′-TGGAGGAATTCTTGCTTTGC-3′	5′-CGTACATGTCAGCCAGCTTC-3′
MMP2	5′-TCTCCTGACATTGACCTTGGC-3′	5′-CAAGGTGCTGGCTGAGTAGATC-3′
MMP9	5′-TTGACAGCGACAAGAAGTGG-3′	5′-GCCATTCACGTCGTCCTTAT-3′

## Supplementary Materials

The following are available online at www.mdpi.com/2072-6694/10/3/68/s1, Figure S1. Real time gene expression studies ER stress markers in TA and Thapsigargin (TG) (Positive control for ER stress) treated cells (*PERK*, *EIF2S1*, *BiP*, *CHOP*, and *ATF4*). The level of significance was represented as ** *p* < 0.01, *** *p* < 0.001. Figure S2. Graphical representation of cells migrated/invaded after TA treatment in prostate cancer cells. The level of significance was represented as ** *p* < 0.01, *** *p* < 0.001. Table S1. Various genes altered during TA treatment (20 µM) in C4-2 and PC-3 prostate cancer cells.
