# Supplementary material for: Tannic Acid Induces Endoplasmic Reticulum Stress-Mediated Apoptosis in Prostate Cancer
Source: Cancers (Basel). 2018 Mar 7;10(3):68. doi: 10.3390/cancers10030068 (PMC5876643; doi:10.3390/cancers10030068)
Supplement: Supplementary file 1 [file cancers-10-00068-s001.pdf]

Supplementary Materials: Tannic Acid Induces Endoplasmic Reticulum Stress-Mediated Apoptosis in Prostate Cancer

Prashanth K.B. Nagesh, Elham Hatami, Pallabita Chowdhury, Vivek K. Kashyap, Sheema Khan, Bilal B. Hafeez, Subhash C. Chauhan, Meena Jaggi and Murali M. Yallapu

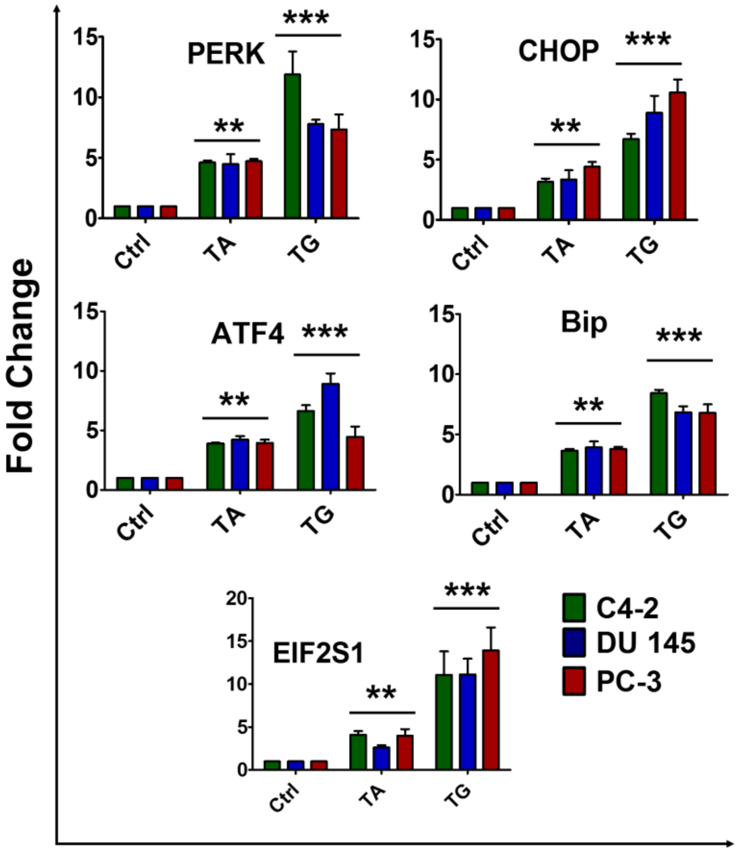

Figure S1. Real time gene expression studies ER stress markers in TA and Thapsigargin (TG) (Positive control for ER stress) treated cells (*PERK, EIF2S1, BiP, CHOP,* and *ATF4)*. The level of significance was represented as ** $p < 0.01$, *** $p < 0.001$.

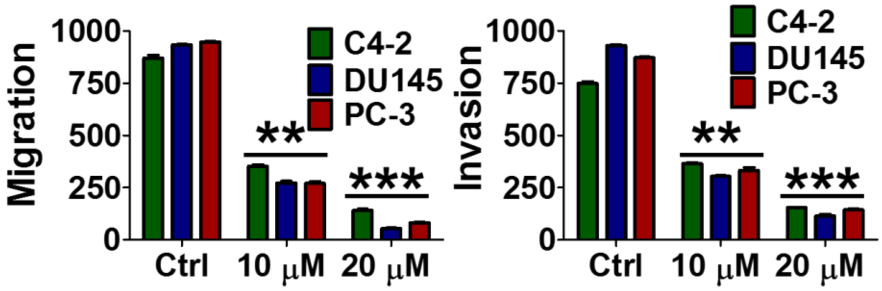

Figure S2. Graphical representation of cells migrated/invaded after TA treatment in prostate cancer cells. The level of significance was represented as ** $p < 0.01$, *** $p < 0.001$.

Table S1. Heatmap values of genes in TA treated C4-2 and PC-3 cells. Various genes altered during TA treatment (20 μM) in prostate cancer cells. The gene fold expression and heat maps were evaluated in the TA treated prostate cancer cells. The fold expression were determined through ratio of intensity values. We have shown the fold expression values for proteins related to ER stress, cell cycle, EMT and Apoptotic signaling in Supplementary Information Table S1. The Heat maps were generated based on the fold expression. The heat maps were generated as described in the manuscript using heatmapper software.

Genes	Cancer cells		Genes	Cancer cells	
	C4-2	PC-3		C4-2	PC-3
ER stress signaling			Cell cycle signaling		
DDIT3	1.470606	1.913439	CDK4	0.697525	0.871108
ATF4	1.617035	1.475808	CDK6	0.430902	0.671971
EIF2AK3	1.878942	1.92969	CCND1	0.64721	0.791913
HSPA5	1.867092	1.910386	CDKN1A	2.138811	1.492021
EIF2S1	1.344201	1.722456	CDKN2C	1.8199	1.497823
ERN1	1.675686	1.828875	RB1	1.990735	1.82538
P4HB	0.879461	0.847845	CDK10	0.481994	0.720143
PPP1R15A	1.37384	2.398293	CDKN1C	1.22931	1.109784
XBP1	0.837196	0.739063	CDK15	0.727125	0.873305
MTOR	0.776442	0.843005	CDK18	0.64683	0.697571
EMT signaling			Apoptotic signaling		
CDH1	1.349361	1.40644	MCL1	0.880897	0.730042
MMP2	0.68989	0.713516	XIAP	0.802588	0.801521
MMP9	0.861321	0.464802	BCL2	0.827866	0.843217
ZEB1	0.396704	0.303452	BIRC5	0.766208	0.878535
TWIST1	0.366947	0.851236	BCL2L1	0.883597	0.634849
SNAI1	0.563721	0.812106	BAX	1.998396	1.852566
CLDN1	1.691079	1.734741	BCL2L11	1.781725	1.555375
OCLN	1.611185	1.393158	BIK	1.797336	1.605881
KRTAP19-6	1.662104	1.445187	BID	1.637967	1.974735
KRT34	1.19368	3.822781	BAD	1.394563	1.21874
KRT1	1.813328	1.649739			

© 2018 by the authors; licensee MDPI, Basel, Switzerland. This article is an open access article distributed under the terms and conditions of the Creative Commons by Attribution (CC-BY) license (http://creativecommons.org/licenses/by/4.0/)
